# Intrauterine Infusion of TGF-β1 Prior to Insemination, Alike Seminal Plasma, Influences Endometrial Cytokine Responses but Does Not Impact the Timing of the Progression of Pre-Implantation Pig Embryo Development

**DOI:** 10.3390/biology10020159

**Published:** 2021-02-17

**Authors:** Cristina A. Martinez, Josep M. Cambra, Xiomara Lucas, Graça Ferreira-Dias, Heriberto Rodriguez-Martinez, Maria A. Gil, Emilio A. Martinez, Cristina Cuello, Inmaculada Parrilla

**Affiliations:** 1 Faculty of Veterinary Medicine, International Excellence Campus for Higher Education and Research “Campus Mare Nostrum”, University of Murcia, 30100 Murcia, Spain; cristina.martinez-serrano@liu.se (C.A.M.); josepmiquel.cambra@lum.es (J.M.C.); xiolucas@um.es (X.L.); mariagil@um.es (M.A.G.); emilio@um.es (E.A.M.); parrilla@um.es (I.P.); 2 Institute for Biomedical Research of Murcia (IMIB-Arrixaca), Campus de Ciencias de la Salud, 30120 Murcia, Spain; 3 Department of Biomedical & Clinical Sciences (BKV), BKH/Obstetrics & Gynaecology, Faculty of Medicine and Health Sciences, Linköping University, SE-581 83 Linköping, Sweden; heriberto.rodriguez-martinez@liu.se; 4 Department of Morphology and Function, University of Lisbon, 1300-477 Lisbon, Portugal; gmlfdias@fmv.ulisboa.pt

**Keywords:** pig, seminal plasma, endometrium, explants, cytokines, maternal immune response, embryo

## Abstract

**Simple Summary:**

Although endometrial immune regulation in pigs during the early preimplantation period is poorly documented, particularly under conditions of embryo transfer (ET), it is recognized that seminal plasma (SP) induces molecular changes in the reproductive tract, influencing numerous reproductive functions. A principal constituent of SP is the cytokine transforming growth factor β1 (TGF-β1), which has an important role in embryo development, pregnancy establishment, and progression. The present study evaluated different intrauterine infusion treatments at estrus (40 mL of SP, porcine TGF-β1 in an extender, or an extender alone (control)) by mimicking an ET scenario in so-called “donor” (inseminated) and “recipient” (uninseminated) sows. We investigated the effects of these treatments on day 6 embryo development (“donors”) and endometrial explants’ cytokine production (“donors” and “recipients”). SP infusion positively influenced embryo development compared with TGF-β1 or extender infusions. Infusion treatments differentially affected endometrial cytokine production, with the effects being stronger in “donors” than in “recipients.” Increased knowledge of the effects of SP or some of its active components on the female immune system may help to develop strategies for increasing the reproductive efficiency for the benefit of pig ET.

**Abstract:**

Seminal plasma (SP) in the female genital tract induces changes that affect multiple reproductive processes. One of the active components in SP is the transforming growth factor β1 (TGF-β1), which has major roles in embryo development and pregnancy. Embryo transfer (ET) technology is welcomed by the pig industry provided that embryo quality at embryo collection as well as the fertility and prolificacy of the recipients after the ET is increased. This study evaluated different intrauterine infusion treatments at estrus (40 mL of SP, TGF-β1 cytokine in the extender, or the extender alone (control)) by mimicking an ET scenario in so-called “donor” (inseminated) and “recipient” (uninseminated) sows. On day 6 (day 0—onset of estrus), all “donors” were laparotomized to determine their pregnancy status (presence and developmental stage of the embryos). In addition, endometrial explants were collected from pregnant “donors” and cyclic “recipients,” incubated for 24 h, and analyzed for cytokine production. SP infusions (unlike TGF-β1 infusions) positively influenced the developmental stage of day 6 embryos. Infusion treatments differentially influenced the endometrial cytokine production, mainly in donors. We concluded that SP infusions prior to AI not only impacted the porcine preimplantation embryo development but also influenced the endometrial cytokine production six days after treatment, both in donors and recipients.

## 1. Introduction

Boar ejaculate contains a large volume of seminal plasma (SP), most of which is removed during sperm collection or diluted for the preparation of artificial insemination (AI) doses. The implementation of the AI technology worldwide was based on excellent reproductive performance, which demonstrated that satisfactory levels of fertility and prolificacy could be achieved with only scarce amounts of SP, at least in pigs. However, it is widely assumed that SP has a key role not only in the transport and survival of spermatozoa [1] but also in subsequent reproductive events [2,3,4]. For instance, in pigs, SP accelerates ovulation [5,6,7], promotes the synthesis of progesterone by supporting the development of corpora lutea [8], and improves reproductive outcomes [9,10,11,12]. The positive effects of SP on pregnancy outcomes have also been reported in other animal species [3,13,14,15] and humans [16,17].

Numerous studies in domestic species, rodents, and humans have shown that SP components are interconnected with the female reproductive tract regarding promoting critical molecular and cellular modifications that influence numerous processes [2,18,19]. Thus, among other actions, the SP promotes the release of cytokines that support the development of preimplantation embryos and the induction of the expression of factors related to angiogenesis and attachment processes, all of which relates to the attainment of the status of maternal immune tolerance to hemiallogeneic embryos [19]. Interestingly, the exposure of the uterus to SP during estrus not only induced endometrial changes in multiple genes and pathways during the periovulation time [20,21] but also modified the endometrial and the blastocyst transcriptomes on day 6 of pregnancy by upregulating numerous genes related to immune pathways, embryonic development, and implantation [22,23]. These and other results [24] clearly demonstrate that the effects of SP infusions during estrus remain influential over time and affect subsequent events related to pre-implantation embryo development and implantation. 

The beneficial effects of SP on reproductive processes are possibly due to the indirect actions of regulatory SP components causing endometrial secretion of embryotrophins. However, the SP molecule(s) responsible for these effects remains unknown. SP is a complex composite fluid that is formed by multiple secretory glands and includes numerous cytokines and growth factors with a broad diversity of biological functions, as well as proteins. An important and abundant compound in SP is TGF-β (transforming growth factor β), principally TGF-β1 [18], which has been found to be involved in the regulation of female immune responses to murine and human SP [25,26]. Moreover, this cytokine increases the production of the granulocyte-macrophage colony-stimulating factor (GM-CSF) in uterine epithelial cells [4], which has key roles in increasing the inner cell mass in mouse embryos in vitro [27] blastocyst development in cattle [28] and humans [29,30], and in establishing pregnancy [31]. For these reasons, it has been suggested that TGF-β may be one of the SP factors involved in immune changes in the porcine uterus [32], although this remains to be validated.

Maternal cytokines, which are mainly produced by the oviductal and endometrial epithelial cells [33], act primarily as regulatory factors in numerous reproductive processes, including maternal immune tolerance to hemiallogeneic embryos, embryo development, appropriate implantation, and normal placental functions [34,35]. The regulation of oviductal and uterine cytokine secretion by the SP has been reported in rodents, humans, and pigs [19]. These studies have indicated that SP factors are interrelated with epithelial cells during the periconception period when stimulating expression of several proinflammatory cytokines [24,26,36,37]. These inflammatory cytokines induce the recruitment of granulocytes to the uterine lumen, which are responsible for a sterile uterine environment, as well as macrophages and dendritic cells that initiate maternal immune tolerance during pregnancy [2]. However, despite the importance of the embryo–maternal dialogue that is mediated by cytokines, little attention has been paid to the effects of SP or some of its active components on maternal cytokine regulation during the subsequent preimplantation periods to conception.

After more than 60 years of difficulties, embryo transfer (ET) technology is ready to be used by the pig industry [38,39]. However, for practical purposes, it would be fundamental to increase embryo quality at the embryo collection, as well as the fertility and prolificacy of the recipients after transfer. To this end, the use of SP or TGF-β1 infusions during estrus could play a key role in improving the efficiency of ET technology. This is of particular relevance not only for donors but also for recipients, which do not have any contact with AI components or embryos before transfer.

Considering its potential relevance in an ET scenario, the aims of this study were to evaluate the effects of SP and TGF-β1 infusions pre-AI on (1) embryo development and viability in pregnant (“ET-donor”) sows, and (2) the cytokine levels of endometrial explants in pregnant (“ET-donor”) and cyclic (“ET-recipient”) sows. We analyzed both parameters on day 6 of the cycle because that day is commonly used for embryo collection and transfer in ET programs. 

## 2. Materials and Methods

### 2.1. Animals

The experiments were performed following the 2010/63/EU directive, being previously accepted by the Murcia Autonomous Government (Murcia, Spain) and by the Ethical Committee for Experimentation with Animals of the University of Murcia (Spain); this study was registered with the research codes 01062016/13160604 and 22072015, respectively.

All the sows included in the study (parity 2 to 7) were weaned at day 21–24 postpartum and were selected according to an optimal reproductive history (the average fertility and litter sizes were >90% and >10 piglets, respectively) and adequate body condition (2.75 to 3.25 on a five-point scale on the day of weaning) following the criteria previously described [40]. Sows belonging to Agropor SA (Murcia, Spain) were individually confined in gestation crates in a building equipped with an automatically operating system that controlled the temperature, ventilation, and humidity. The AI doses were obtained from fertile Duroc boars (2 to 3 years old) held in individual ventilation- and temperature-controlled pens at a pig AI center (AIM Iberica, Murcia, Spain). The animals had ad libitum access to water and were fed regular commercial diets.

### 2.2. Experimental Design

Fifty-one sows were intrauterinely infused with 40 mL of SP (*n* = 19), porcine TGF-β1 (3 ng/mL) in a Beltsville Thawing Solution (BTS) extender [41] (*n* = 16), or BTS alone (control group; *n* = 16) 30 min before each of two postcervical AIs. All these sows were considered as embryo “donors” within an eventual embryo transfer (ET) situation. In addition, a total of nine non-inseminated sows were distributed into the above-listed groups (three sows per group). These sows were considered as cyclic “recipients” within an ET scenario.

On day 6 (day 0—onset of estrus), all “donors” were surgically intervened to determine their pregnancy status (presence and developmental stage of the embryos). In addition, endometrial samples were collected from 18 pregnant “donors” and cyclic “recipients” to determine the changes in cytokine concentrations. The endometrial samples were taken from three different sites of each uterine horn (six samples per sow) and processed as described below. 

### 2.3. Superovulation and the Detection of Estrus

Twenty-four hours after weaning, those sows considered as embryo “donors” were superovulated with 1000 IU equine chorionic gonadotrophin (eCG; 24 h postweaning), followed by 750 IU human chorionic gonadotrophin at the onset of estrus (only in sows in estrus at 72–96 h post-ECG), as previously described [22].

The detection of estrus was performed twice-daily, beginning on the day following weaning and was carried out by allowing snout-to-snout contact of sows with mature boars and manually applying back-pressure. The onset of estrus was identified when the animals first displayed a clear standing reflex (day 0). 

### 2.4. Insemination of “Donors”

Sows grouped as “donors” were inseminated (postcervical AI) at 6 h and 24 h after the beginning of estrus with AI sperm doses containing 1.5 × 10^9^ spermatozoa that were extended to 40 mL with BTS. Within each replicate, a comparable number of sows (*n* = 3) from each of the three groups was inseminated with pooled sperm doses from the same three boars. The AI doses were kept at 18 °C for a maximum of 24 h.

### 2.5. Preparation of Sperm-Free Seminal Plasma

Sperm-free SP was exclusively obtained from entire gel-free ejaculates from 14 boars (3 ejaculates/boar) using a semi-automatic semen collection system (Collectis^®^, IMV Technologies, L’Aigle, France). The ejaculates were sent to the laboratory within a maximum of 2 h postcollection, where they were centrifuged three times (10 min at 1500× *g*) at 17 °C. In all cases, the absence of spermatozoa in the final supernatant was microscopically verified. The SP from at least seven boars was pooled, aliquoted into individual AI bottles (40 mL SP per bottle), and kept at −20 °C until use. Before the inseminations, the SP doses were thawed (37 °C, 20 min) and introduced with a postcervical catheter into the uterine body of each sow.

### 2.6. Collection and Evaluation of the Embryos

Each animal was subjected to a laparotomy on day 6. Sedation was induced using azaperone (intramuscular (i.m.); 2 mg/kg of body weight; Stresnil^®^, Landegger Strasse, Austria). General anesthesia was initiated 10 min later with sodium thiopental (intravenous (i.v.); 7 mg/kg of body weight; B. Braun Vet Care SA, Barcelona, Spain) and maintained with isoflurane (3.5–5%; IsoFlo^®^, Madrid, Spain). 

Embryo collection was performed as previously reported [42]. Briefly, after counting the number of corpora lutea on each ovary, the embryos were recovered by washing the tip of each uterine horn with 30 mL of Tyrode’s lactate–HEPES–polyvinyl alcohol medium [43]. Then, the embryos were assessed for quality and development. Embryos with appropriate morphologies and development following the norms of the International Embryo Transfer Society [44] were considered viable. The developmental stages of the viable embryos were scored for statistical analysis, as previously described [45]: 1—compacted morulae (unidentifiable blastomeres in the periphery), 2—early or full blastocysts (embryos with an initial or easily visible blastocoel and an inner cell mass and discernible trophoblast), and 3—expanded or hatching/hatched blastocysts (blastocysts with an increased diameter and thinned, broken, or lost zona pellucida). 

### 2.7. Endometrial Tissue Collection

Some (*n* = 3) pregnant “donors” from each treatment group were hysterectomized on day 6 immediately following the embryo collection. Hysterectomies in all “recipient” females were also performed on day 6 of the cycle. Endometrial samples from the antimesometrial distal (next to the uterine body), middle, and proximal (next to the uterotubal junction) zones of each uterine horn were obtained from each sow.

### 2.8. Endometrial Explant Culture

Endometrial strips with evident epithelial lining were transferred into a culture medium (NCSU-23) [46], cut into smaller pieces (2–3 mm^3^), and washed in a phosphate-buffered saline medium containing 20 μg/mL gentamicin. Three endometrial pieces (40 mg/mL) were transferred into the wells of a 12-well plate with 3 mL culture medium per well. These explants were preincubated at 38 °C in 5% CO_2_ in air with agitation at 150 rpm for 1 h and, after replacing the culture medium, further incubated for 22 h. Samples from each uterine location (proximal, middle, and distal for each uterine horn; six samples per sow in total) were cultured in triplicate, and the conditioned media collected from each uterine location was pooled and centrifuged at 13,000× *g* for 5 min to eliminate debris. Supernatants were kept at −80 °C until cytokine analysis. The time point of 22 h was chosen for being within the interval when the innate immune response occurs. Explant integrity was assessed by measuring the lactate dehydrogenase (LDH) activity in harvested supernatants at 0 h and 22 h of culture using an analyzer (AU 600, Olympus, Minneapolis, MN, USA). The mean values of LDH activity at 0 h and 22 h of the culture were 2.0 ± 0.8 mU/mg and 7.1 ± 2.3 mU/mg, respectively. 

### 2.9. Cytokine Analysis

The presence and concentrations of 16 cytokines/chemokines in culture media samples following incubation with endometrial samples from the different experimental groups were determined using Luminex xMAP^®^ technology (Luminexcorp, Austin, TX, USA). Two porcine specific cytokine pre-coated magnetic bead kits, PCYTMG-23k-13PX and TGFβ-64K-03 (Merck Chemicals and Life Science SA, Madrid, Spain), were used, which allowed for the simultaneous quantification of 13 cytokines (GM-CSF, interferon gamma (IFNγ), interleukin (IL)-1α, IL-1ra, IL-1β, IL-2, IL-4, IL-6, IL-8, IL-10, IL-12, IL-18, and tumor necrosis factor α (TNFα)) and 3 cytokines (TGF-β1, TGF-β2, and TGF-β3), respectively. The recommended manufacturer’s instructions were followed for both kits. 

Cytokine standard curves containing seven (PCYTMG-23k-13PX) or six (TGFBMAG-64K-03) standard points were created for each cytokine. For the quantification of TGF-β cytokines, the spent medium was acidified with HCl and diluted 1:30 with sample diluent to adjust to the standard curve range. The serum matrix included in the kits was used to imitate the composition of the spent medium samples in the standard, control, and blank measurements. Two controls included in the kits were added as singlets to their corresponding wells. Immediately after sonication, the bead solution was added to each well for incubation (18 h) at 4 °C in the dark. Then, the plate was emptied using a multiscreen vacuum manifold and washed two or three times (depending on the kit) with the washing solution included in the kits. After washing, the detection antibody was added to each well. The plates were incubated at room temperature in darkness for 60 min or 120 min (depending on the kit), followed by streptavidin–phycoerythrin addition and further incubation for 30 min at room temperature. The plates were washed and run on a MAGPIX R (Luminexcorp, Austin, TX, USA). Data acquisition and data analysis were performed with the xPONENT software version 4.2 (Luminexcorp, Austin, TX, USA) and MILLIPLEX R Analyst Version 5.1 (Merck Millipore, Darmstadt, Germany), respectively. The median fluorescent intensity was analyzed using a five-parameter logistic curve fit to determine the concentrations (pg/mL) of the cytokines in the samples. 

### 2.10. Statistical Analysis

The data were statistically analyzed with SPSS Statistics version 19 (IBM SPSS Statistics, Chicago, IL, USA). Continuous variables were analyzed using the Shapiro–Wilk test for normality and variables with a normal distribution (number of corpora lutea, number of viable embryos, and recovery and fertilization rates) were compared using ANOVA. The ANOVA model included the fixed effect of the group and the random effect of the replicate (six replicates). When the ANOVA showed a significant effect, the means were analyzed using Bonferroni’s test. The data of the embryo scoring for the developmental stage and the cytokine values were compared using the non-parametric Kruskal–Wallis test followed by the Mann-Whitney U post hoc test. The concentrations of IL-10 in some of the “recipient” samples (28 out of 54) were below the quantitation limit for analysis. To avoid any bias, these concentrations were replaced with a value corresponding to half of the limit of detection, which was 8 pg/mL. Outliers were identified via boxplots and eliminated from the statistical analysis. A *p*-value < 0.05 was considered to be statistically significant. The data are shown as the means ± SD, medians, and percentiles.

## 3. Results

### 3.1. Reproductive Parameters

Forty-seven out of the 51 “donors” (92.1%) had embryos at the collection time, with no differences between the treatment groups. All sows with embryos had more than eight viable embryos in each uterine horn and were therefore included in the study. Table 1 provides the results regarding the reproductive performance of “donors” according to the infusion treatments prior to AI. No differences were observed between groups for the number of corpora lutea, recovery rates, number of viable embryos, or fertilization rates. The percentages of embryos staging morula, blastocysts, or expanded, hatching/hatched blastocysts at collection were 27.0%, 48.4%, and 24.6%, respectively. This embryo developmental distribution was affected by the SP-infusion treatment. The sows infused with SP had fewer morulae (9.9%) and more embryos in the blastocyst stages (90.1%) compared to the other groups (≈30% and ≈70% for the morulae and blastocyst stages, respectively) (*p* < 0.001). These differences resulted in an increase in the embryo developmental stage score (Table 1).

### 3.2. Cytokine Production by Endometrial Explants 

In this study, we evaluated the cytokine profiles secreted by endometrial explants from “donor” and “recipient” sows and how these profiles were influenced by SP or TGF-β1 infusions prior to AI. Since there were no significant differences in the cytokine levels between the endometrial explants collected from different uterine regions (distal, middle, and proximal) or uterine horns (right and left), the cytokine concentrations were averaged for each sow within each treatment, regardless of the site of the endometrial origin.

#### 3.2.1. Effect of SP and TGF-β1 Infusions Prior to AI on Cytokine Production by Endometrial Explants of “Donor” Sows 

**Figure 1:**
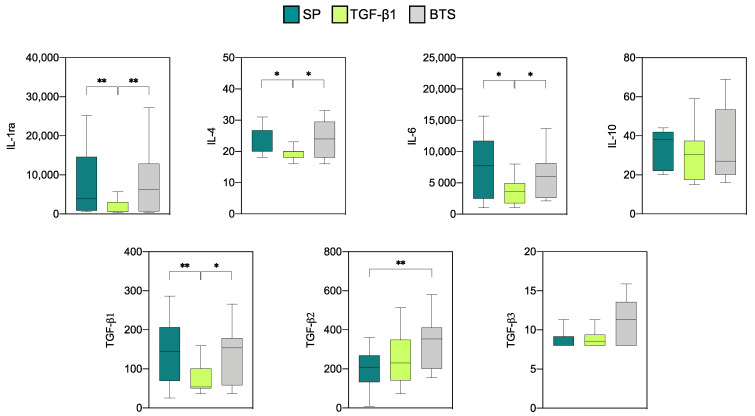
Levels of anti-inflammatory cytokines (pg/mL) on day 6 of pregnancy in endometrial explants of “donor” sows infused with 40 mL of SP, TGF-β1 in BTS extender (TGF-β1), or BTS alone (control) 30 min prior to each of two inseminations. The box plots show the median (line) and the interquartile ranges (boxes, Q1–Q3). * *p* < 0.05, ** *p* < 0.01.

Analysis of the expression of seven anti-inflammatory cytokines by endometrial explants from “donors” showed that all the cytokines were produced at measurable levels (Figure 1). The concentrations of IL-1ra and IL-6 were the highest among the seven cytokines, followed by TGF-β1 and TGF-β2. For the three remaining cytokines, namely, IL-4, IL-10, and TGF-β3, low levels were detected. The infusion treatments had a significant effect on the levels of all anti-inflammatory cytokines analyzed, except for IL-10 and TGF-β3. The levels of IL-1ra, IL-4, IL-6, and TGF-β1 in the SP and control groups were similar and were significantly higher than those obtained from the TGF-β1 group (Figure 1). The concentration of TGF-β2 was significantly lower in the SP group only when compared with the control group (*p* < 0.01). 

**Figure 2:**
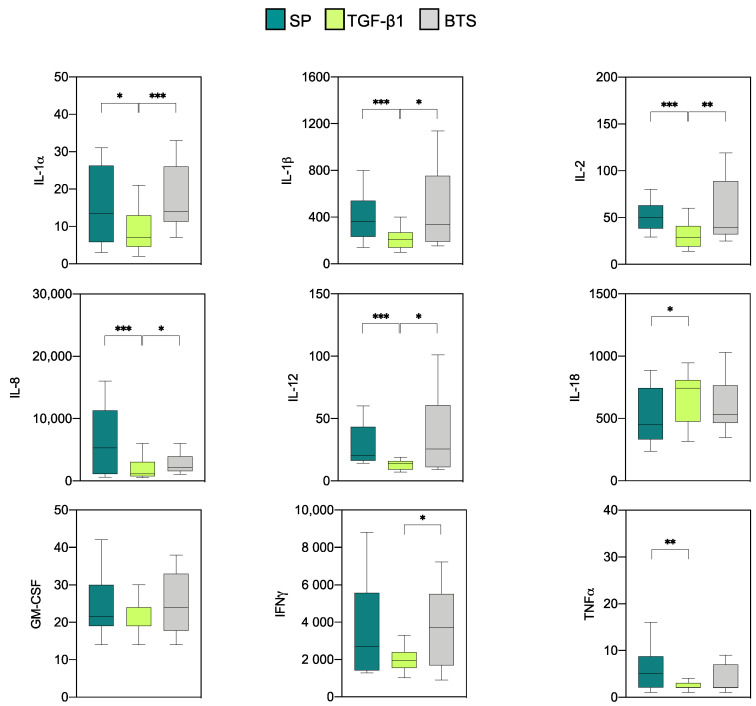
Levels of proinflammatory cytokines (pg/mL) on day 6 of pregnancy in endometrial explants of “donor” sows infused with 40 mL of SP, TGF-β1 in BTS extender (TGF-β1), or BTS alone (control) 30 min prior to insemination. The box plots show the median (line) and the interquartile ranges (boxes, Q1–Q3). * *p* < 0.05, ** *p* < 0.01, *** *p* < 0.001.

Measurable levels of the nine pro-inflammatory cytokines analyzed were obtained in all experimental groups (Figure 2). Relatively high concentrations of IL-8 and IFNγ were detected regardless of the experimental group, while IL-1β and IL-18 displayed moderate concentrations and the rest of the proinflammatory cytokines displayed low relative concentrations. The infusion with TGF-β1 led to a decreased (*p* < 0.05) concentrations of IL-1α, IL-1β, IL-2, IL-8, and IL-12 compared with the rest of the groups. Similarly, the TGF-β1 group had lower IFNγ values only when compared with the control group (*p* < 0.05), and lower TNFα values in comparison with those obtained for the SP (*p* < 0.01) group. IL-18 was unique in that it had higher levels in the TGF-β1-treated samples compared to the SP (*p* < 0.05) group. 

#### 3.2.2. Effect of SP and TGF-β1 Infusions Prior to AI on Cytokine Production by Endometrial Explants of “Recipient” Sows

**Figure 3:**
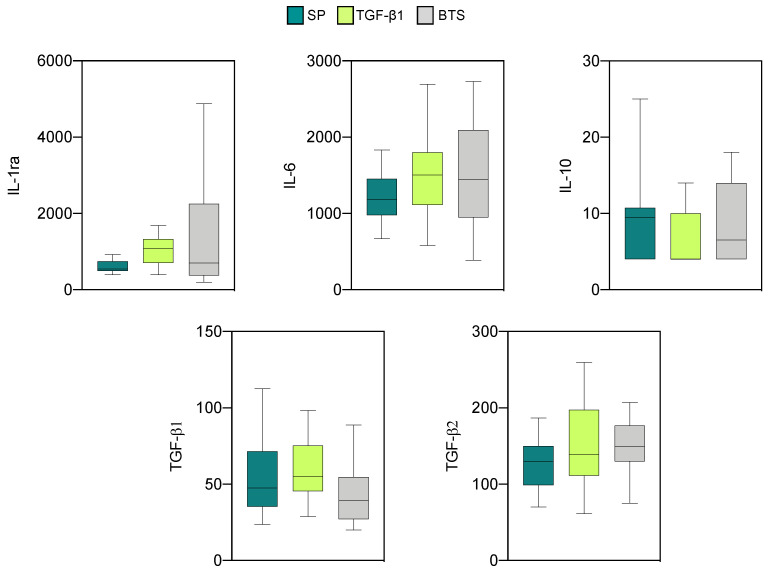
Levels of anti-inflammatory cytokines (pg/mL) on day 6 of the cycle in endometrial explants of “recipient” sows infused with 40 mL of SP, TGF-β1 in BTS extender (TGF-β1), or extender alone (BTS). These sows were not inseminated. The box plots show the median (line) and the interquartile ranges (boxes, Q1–Q3).

**Figure 4:**
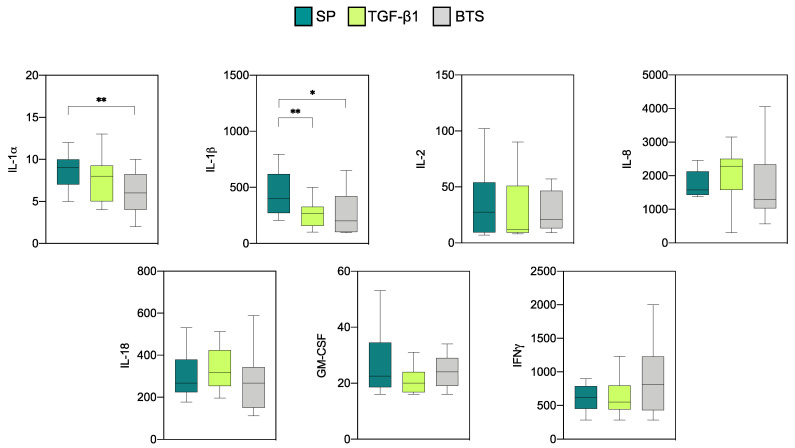
Levels of proinflammatory cytokines (pg/mL) on day 6 of the cycle in endometrial explants of “recipient” sows infused with 40 mL of SP, TGF-β1 in BTS extender (TGF-β1), or extender alone (BTS) 30 min prior to insemination. These sows were not inseminated. The box plots show the median (line) and the interquartile ranges (boxes, Q1–Q3). * *p* < 0.05, ** *p* < 0.01.

The profiles of anti- and proinflammatory cytokines in endometrial explants of “recipients” are shown in Figure 3 and Figure 4, respectively. Detectable values were obtained for five anti-inflammatory cytokines (IL-1ra, IL-6, IL-10, TGF-β1, and TGF-β2). The concentrations of the remaining anti-inflammatory cytokines were too low to be detected and were not analyzed.

The levels of IL-1ra and IL-6 were the highest among the five cytokines evaluated. The infusion treatments did not influence the levels of any of these cytokines. Regarding the proinflammatory cytokines, measurable levels were detected for all cytokines (IL-1α, IL-1β, IL-2, IL-8, IL-18, GM-CSF, and IFNγ), except IL-12 and TNFα, which displayed levels below the detection limits and were not further analyzed. High concentrations of IL-8 and IFNγ and intermediate concentrations of IL-1β and IL-18 were observed in all experimental groups. The rest of the proinflammatory cytokines displayed low relative concentrations. Significant differences between the groups were only observed for IL-1α and IL-1β. Specifically, SP infusions induced higher levels (*p* < 0.01) of IL-1α when compared with the control group. SP infusions also increased (*p* < 0.05) the level of IL-1β compared to the rest of the groups.

#### 3.2.3. Comparison of Cytokine Profiles between the “Donor” and ”Recipient” Sows

**Figure 5:**
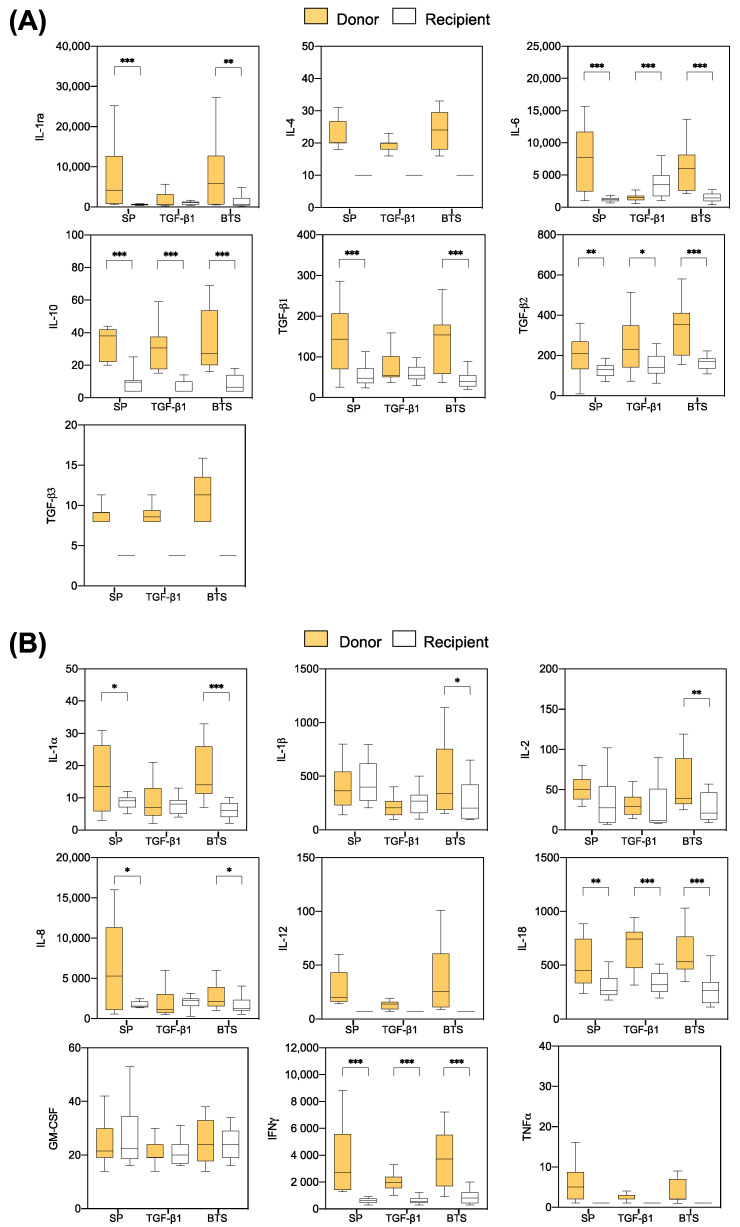
Differences found six days after the onset of estrus in endometrial anti-inflammatory (**A**) and pro-inflammatory (**B**) cytokine levels between “donors” and “recipients” infused with 40 mL of SP, TGF-β1 in BTS extender (TGF-β1), or BTS alone (control) 30 min prior to insemination. The box plots show the median (line) and the interquartile ranges (boxes, Q1–Q3). * *p* < 0.05, ** *p* < 0.01, *** *p* < 0.001.

Most of the anti- and proinflammatory cytokines were produced at higher levels in explants from “donor” sows compared to those of “recipient” sows, regardless of the experimental group. The anti-inflammatory cytokines IL-6, IL-10, and TGF-β2 displayed differences (*p* < 0.05) between the “donor” and “recipient” sows in all treatments; for IL-1ra and TGF-β1, differences were significant in all groups, except the TGF-β1 group (Figure 5A). Regarding the pro-inflammatory cytokines, the levels of IL-18 and IFNγ were higher (*p* < 0.01) in “donors” than in “recipients” in all treatment groups. In addition, the levels of IL-1α and IL-8 were different (*p* < 0.05) between the “donor” and “recipient” sows in all groups, except the TGF-β1 group (Figure 5B). 

## 4. Discussion

The present study showed that, under the devised experimental design, neither TGF-β1 nor BTS (control) infusion treatments prior to AI affected embryo development. However, SP infusions positively influenced the developmental stage of day 6 embryos. We demonstrated for the first time that infusion treatments affected the cytokine production profile of endometrial explants from day 6 sows, mainly in sows considered as “donors” in the scenario of ET practice. In addition, we report important differences in the endometrial cytokine production between “donor” and “recipient” sows, regardless of the infusion treatment.

While infusion treatments prior to AI did not affect the fertilization or survival rates of day 6 embryos, SP-infusions advanced the timing of embryo development compared to that observed after TGF-β1 or BTS infusions, which is an important biological indicator of embryo quality [47]. These findings confirmed previous studies indicating that the effect of SP administration at estrus is not only immediate [20,21] but also remains during the preimplantation time [22,24] and modifies the timing of embryo development [22]. Although the advanced embryo development detected in the SP group might be related to the influence that SP has on the time of ovulation [5,6,7], recent studies suggest that the timing of embryo development is controlled and modified at the molecular level by a possible direct effect of SP [22,23]. First, SP infused during estrus modifies the endometrial transcriptome on day 6 of gestation, upregulating the genes and pathways that are closely related to embryo development and maternal immune system tolerance [22]; second, such SP infusions also upregulate the expression of genes and pathways in day 6 blastocysts that are linked to processes that control embryonic development, implantation, or the progression of pregnancy [23]. Altogether, these molecular modifications might modulate the endometrial secretions of embryotrophic molecules that protect and nurture the conceptuses during its free-floating period prior to implantation.

The results of the present study show that factors other than TGF-β1 are responsible for the impact that the SP has on embryo development. We speculate that the lack of a TGF-β1 effect on embryonic development could be due to the concentration used since a dose–effect study was not performed. However, the sows from the TGF-β1 group were infused with 3 ng/mL of TGF-β1, which mimicked the physiological concentration of the cytokine in the boar ejaculate [48,49] and, therefore, its theoretical concentration in the SP-group (a group in which the effects of SP on embryonic development were evident). It is also likely that TGF-β1 alone failed to induce these beneficial effects because, as with many cytokines, TGF-β1 may need to act in synergy with other cytokines to properly exert its functions [32], and therefore, the positive effects of SP infusions on embryo development may be mainly due to the concerted action of many components present in the SP and not to the effects of a single particular cytokine. Supporting this, while the addition of SP to AI doses increased pregnancy outcomes [12], the addition of TGF-β1, even at higher concentrations than those used herein, did not influence the implantation success [50]. Finally, the lack of effect of TGF-β1 on embryo development could be related to the different biological effects of the TGF-β1 forms present in SP and the purified soluble active form used in our study. Along this line, an inappropriate targeting of this soluble active form failed to mimic the effects of naturally activated TGF-β molecules at the tissue level [51].

The pig endometrium is influenced by the presence of SP, sperm, and extender, and the uterine environment can change in response to these insemination components [24,52,53]. Our results further indicate that cytokine production by endometrial explants was affected by the different infusion treatments administered prior to AI and that different patterns of expression between experimental groups were detected as far out as 6 days after the treatments. These results were not unexpected, as SP infusions pre-AI also altered the transcriptome of the endometrium and blastocysts six days later [22,23]. 

In “donor” sows, endometrial explants from SP and BTS (control) groups displayed similar patterns of cytokine production, except for TGF-β2. In contrast, other studies have reported variations in the cytokine expression between SP and extender groups, with an increase or decrease of some cytokines on days 5–6 after treatment [24,54]. The discrepancies between studies were likely due to the differences in methodology protocols, measurements of cytokine expression, and types of samples evaluated (endometrial-only explants, or total uterine tissue). In our study, the comparable cytokine production between SP and BTS (control) groups could be because, after BTS infusion, the sows were inseminated with AI doses containing 10% homologous SP, sperm, and extender. These male factors could have counteracted the known reactive effect of the extender [12] and as a result, the response between SP and BTS groups was comparable. However, because an improvement in embryo development was observed in the SP “donors” compared to the BTS “donors,” there must be other mechanisms triggered by SP beyond the response at the cytokine production level that modulate the timing of uterine events favoring embryo development and pregnancy establishment, as previously suggested [3,55]. 

It is known that the TGF-β cytokine family plays a major role in the immunomodulatory effects of SP on the maternal genital tract that lead to a preimplantation immunotolerant environment, which is crucial for the establishment of pregnancy [56]. Moreover, it has been reported that exogenous TGF-β enhances human embryo development in vitro by increasing the number of blastomeres and promoting blastocyst proliferation [57]. In our study, we included TGF-β1 infusions to evaluate their potential for creating an optimal uterine environment for embryo development. The results obtained in endometrial explants from “donor” sows infused with TGF-β1 showed a cytokine profile that was totally different from those in the other groups, with a decrease in most of the cytokines analyzed. As mentioned above, the use of an inappropriate concentration of TGF-β1, the lack of interaction between TGF-β1 and other cytokines, and/or other factors related to the correct functioning of the soluble form of TGF-β1 used could have caused the failure of this treatment to create a favorable endometrial environment for embryo growth. Interestingly, TGF-β1 infusions had no deleterious effects on embryo features, which support previous results found after the supplementation of AI doses with TGF-β1 [50]. 

The differences in cytokine expression between the infusion groups were more evident in “donors” than in “recipients,” with a higher number of both types of cytokines (anti- and proinflammatory) that were significantly increased in the “donors.” The reasons for these differences between the “donors” and “recipients” are unclear. However, following the initial infusion stimulation, there were important differences. “Donors,” but not “recipients,” were subjected to two inducing stimuli (AI components and embryos). These stimuli are able to modify the endometrial immune response in gilts [54] and sows [58]. Moreover, the maternal immune system might detect the presence of pre-implantation embryos and react accordingly [59,60,61]. Our results indicate that despite an initial stimulation, in “recipient” sows, the lack of an AI component stimulus, together with the absence of preimplantation embryos, might have caused a decreased immune response, which resulted in a reduction in cytokine production in the endometrium, with a minor influence from the infusion treatments. 

## 5. Conclusions

Our results indicate that SP infusions prior to AI positively impacted the timing of porcine preimplantation embryo development and that TGF-β1, under the conditions used in this study, was apparently not responsible for this impact. This study demonstrated that the different infusion treatments used before AI differentially influenced the endometrial cytokine production six days after treatment. In “donor” sows, while SP and BTS infusions showed comparable endometrial cytokine production, TGF-β1 infusions displayed a decreased endometrial production of most of the cytokines analyzed. These differences among the experimental groups were more marked in “donors” than in “recipients,” with a greater number of cytokines affected in the “donors.” Whether the infusion of SP or some of its components in “recipient” sows during estrus could help to increase the endometrial receptivity to the allogeneic embryos and to improve their survival and development after ET need to be further elucidated.

## Figures and Tables

**Table 1:** Reproductive parameters in pregnant “donors” obtained 6 days after the infusions of seminal plasma (SP), Beltsville Thawing Solution (BTS) extender containing 3 ng/mL of porcine TGF-β1 (TGF-β1), or BTS alone (control) 30 min prior to insemination.

Treatment	Sows (*n*)	Corpora Lutea	Recovery Rate (%)	Viable Embryos	Fertilization Rate (%)	Embryonic Stage ^1^
SP	18	24.1 ± 8.4	93.3 ± 11.0	20.1 ± 7.4	89.1 ± 19.3	2.3 ± 0.7 ^a^
TGF-β1	14	23.7 ± 5.3	92.9 ± 12.1	19.9 ± 6.0	90.4 ± 14.2	1.8 ± 0.6 ^b^
BTS	15	23.5 ± 6.1	93.2 ± 11.5	21.3 ± 6.4	91.5 ± 14.8	1.9 ± 0.7 ^b^

^1^ The developmental stage was scored from 1 to 3 as follows: 1—morulae, 2—early or full blastocysts, and 3—expanded, hatching, or hatched blastocyst. ^a,b^ Different letters in the same column indicate significant differences (*p* < 0.001). Values are expressed as the mean ± SD (six replicates).

## Data Availability

The data presented in this study are available on reasonable request from the corresponding author.
